# A Virtual Emergency Telemedicine Serious Game in Medical Training: A Quantitative, Professional Feedback-Informed Evaluation Study

**DOI:** 10.2196/jmir.3667

**Published:** 2015-06-17

**Authors:** Iolie Nicolaidou, Athos Antoniades, Riana Constantinou, Charis Marangos, Efthyvoulos Kyriacou, Panagiotis Bamidis, Eleni Dafli, Constantinos S Pattichis

**Affiliations:** ^1^ Department of Communication and Internet Studies Cyprus University of Technology Limassol Cyprus; ^2^ STREMBLE Ventures Ltd Limassol Cyprus; ^3^ University of Cyprus Department of Computer Science Nicosia Cyprus; ^4^ Cyprus Εmergency and Pre-hospital care Association, KSEPA Nicosia Cyprus; ^5^ Department of Computer Science University of Cyprus Nicosia Cyprus; ^6^ Department of Computer Science & Engineering Frederick University Limassol Cyprus; ^7^ Lab of Medical Physics Medical School Aristotle University of Thessaloniki Thessaloniki Greece

**Keywords:** telemedicine, emergency telemedicine, serious games, virtual patients, medical education, professional feedback-informed evaluation, emergency assessment and management

## Abstract

**Background:**

Serious games involving virtual patients in medical education can provide a controlled setting within which players can learn in an engaging way, while avoiding the risks associated with real patients. Moreover, serious games align with medical students’ preferred learning styles. The Virtual Emergency TeleMedicine (VETM) game is a simulation-based game that was developed in collaboration with the mEducator Best Practice network in response to calls to integrate serious games in medical education and training. The VETM game makes use of data from an electrocardiogram to train practicing doctors, nurses, or medical students for problem-solving in real-life clinical scenarios through a telemedicine system and virtual patients. The study responds to two gaps: the limited number of games in emergency cardiology and the lack of evaluations by professionals.

**Objective:**

The objective of this study is a quantitative, professional feedback-informed evaluation of one scenario of VETM, involving cardiovascular complications. The study has the following research question: “What are professionals’ perceptions of the potential of the Virtual Emergency Telemedicine game for training people involved in the assessment and management of emergency cases?”

**Methods:**

The evaluation of the VETM game was conducted with 90 professional ambulance crew nursing personnel specializing in the assessment and management of emergency cases. After collaboratively trying out one VETM scenario, participants individually completed an evaluation of the game (36 questions on a 5-point Likert scale) and provided written and verbal comments. The instrument assessed six dimensions of the game: (1) user interface, (2) difficulty level, (3) feedback, (4) educational value, (5) user engagement, and (6) terminology. Data sources of the study were 90 questionnaires, including written comments from 51 participants, 24 interviews with 55 participants, and 379 log files of their interaction with the game.

**Results:**

Overall, the results were positive in all dimensions of the game that were assessed as means ranged from 3.2 to 3.99 out of 5, with user engagement receiving the highest score (mean 3.99, SD 0.87). Users’ perceived difficulty level received the lowest score (mean 3.20, SD 0.65), a finding which agrees with the analysis of log files that showed a rather low success rate (20.6%). Even though professionals saw the educational value and usefulness of the tool for pre-hospital emergency training (mean 3.83, SD 1.05), they identified confusing features and provided input for improving them.

**Conclusions:**

Overall, the results of the professional feedback-informed evaluation of the game provide a strong indication of its potential as an educational tool for emergency training. Professionals’ input will serve to improve the game. Further research will aim to validate VETM, in a randomized pre-test, post-test control group study to examine possible learning gains in participants’ problem-solving skills in treating a patient’s symptoms in an emergency situation.

## Introduction

### Background

New media technologies such as serious games involving virtual patients align with medical students’ preferred learning styles and are seen positively by them [1-3] as an innovative way for learning skills that are necessary in the medical profession. This is mostly because of the affordances of serious games and virtual patients in engaging learners while at the same time avoiding the risks associated with real patients. Most studies involving virtual patients in serious games in medical education focus on the description of the design and development of innovative ways to teach doctors and medical students [4-8]. However, there is evidence from controlled trial designs or similar rigorous methodologies [9-15] that indicates the effectiveness of proposed serious games and virtual patient interventions in medical education. Not many serious games exist that focus on supporting medical students’ skills in responding to medical emergency situations to manage the symptoms of patients facing cardiovascular complications through telemedicine. Moreover, there is a lack of evaluation/impact measurements with actual professional groups, as most studies that aimed to evaluate serious games and virtual patients had students as participants [1,2,5]. The present study attempted to fill these two gaps with the design and development, and attempt to quantify the potential of a virtual emergency telemedicine serious game in medical training through a professional feedback-informed evaluation study, with 90 professional ambulance crew nursing personnel specializing in the assessment and management of emergency cases.

### Medical Students’ Attitudes Toward Games

Serious games align with digital natives’ [16] preferred learning styles and this seems to apply in medical education as well [17,18]. In the domain of medical education, several studies showed that medical students’ attitudes toward games in medical education, and toward learning from new media, such as immersive 3D virtual environments or virtual mentoring systems, are favorable [1-3]. In a recent study with 217 medical students from two US universities [1], the vast majority (98%) of students liked the idea of using technology to enhance health care education, felt that education should make better use of new media technologies (96%), and believed that games can have an educational value (80%). The attitudes of doctors in training toward virtual mentoring systems were also reported to be favorable in the study of Jaffer et al [3], which examined the effect of a short introduction on how to use virtual systems on 57 junior doctors in the United Kingdom. Evidence for medical students’ preferences in learning from new media, such as immersive 3D virtual environments also comes from a study with 90 second-year Master’s students in pharmacy [2]. The attitudes of 62 medical students were also favorable on the use of serious games’ interactive algorithms involving electronic virtual patients in medical education, as they found the interactive algorithms were effective learning tools, facilitating enhanced knowledge in the field of acute medicine [19].

Ample evidence indicates that serious games align with medical students’ preferred learning styles; therefore, a next step is to evaluate whether empirical evidence, documenting learning gains of such games, also supports their integration in medical education, as it was suggested by [20] in the area of emergency medicine in particular.

### Serious Games Involving Virtual Patients in Medical Education

Serious games development and implementation for medical education is a growing domain. According to Graafland et al [21]*,* who conducted a systematic review of serious games for medical education and surgical skills training, which included 25 research studies and covered 30 serious games published between 1995 and 2012, serious games form an innovative approach toward the education of medical professionals. Serious games attempt to deliver affordable, accessible, and usable interactive virtual worlds, supporting applications in training and education [9]. Following these trends, traditional instructor-centered teaching is yielding to a learner-centered model that puts learners in control of their own learning in medical education [22].

Some of the attributes of games involving virtual patients in medical education that make them attractive and useful include the fact that game environments provide a safe and controlled setting within which players can learn in an engaging way, while avoiding the risks associated with real patients [4]. Virtual patient simulations have the significant advantages of requiring fewer personnel and resources, being accessible at any time, and being highly standardized [23], and may support learning processes and be a valuable complement in teaching communication skills, patient-centeredness, clinical reasoning, and reflective thinking [24]. According to the systematic review by Ghanbarzadeh et al [25], virtual patients can be used by trainees such as nurses, surgeons, students, and other medical staff, and their performance can be assessed and benchmarked in different ways. However, virtual patients are notoriously difficult and costly to author, adapt, and exchange. Historically, this has limited their uptake and utility, despite their being able to provide high-quality learning opportunities [26] and despite enthusiasm about the educational potential of three-dimensional virtual worlds and virtual patients for medical educators [27].

There are many recent examples of studies that involved virtual patients in serious games for medical education in areas such as cardiology for practicing doctors [4], rehearsing professional behaviors, such as taking a patient’s history for medical students [5], improving the efficiency of junior doctor training through a junior doctor medical simulator [6], teaching insulin therapy for primary care physicians [7], surgical training and developing clinical skills to respond to injuries sustained during catastrophic incidents [8], teaching about medical ethics, medical law, and medical professionalism [28], and repurposing Web-based virtual patients to multi-user virtual environments for undergraduate dental education [29]. Of special interest for this study are virtual patient games on emergency treatment in cardiology, which are discussed next.

In the area of cardiology but not focusing on emergency treatment, Dafli, Bamidis, and Dombros [4] described the design and implementation of a pilot scenario with a simulated virtual patient for potential use in Greek medical education. In their scenario, the practicing doctor could interact with a virtual patient to examine him, select diagnostic tests and inquiry methods, select different approaches, and reach a decision with respect to the treatment of a cardiological incident. The content was realistic and it was enhanced with the addition of audiovisual material to simulate medical reality and allow users to develop clinical skills through the system. Even though the application of the virtual patient has not yet been evaluated by a large number of users, it appears to be a promising application that can extend the users’ experience with real patients and allow them to practice their clinical skills in a systematic, safe, and protected way, adjusted to their own needs and level of experience [30].

In the area of emergency treatment but not focusing on cardiology, the University of Auckland’s Second Life simulation island, Medical Centre, and Emergency Room simulations represent a case where more experiential and immersive multi-user virtual environments have been used for designing and hosting virtual patients in serious games and experiential learning tools in emergency medicine and care [31]. Another case is the use of the Second Life virtual simulation environment for mock oral emergency medicine examinations targeting emergency medicine residents, who have the requirement for board certification in order to become emergency physicians [32].

A common theme identified in the literature of studies that involved virtual patients in serious games for medical education is that even though they provide indications that virtual world medical simulations have the potential to enable students to practice professional behaviors in a risk-free environment, providing opportunities for skills practice prior to real-world patient encounters, their work is not always validated through empirical research. This is true for studies such as the one by Danforth et al [5]. The same applies for Diehl et al [7], who plan to evaluate their game, InsuOnline, using a randomized controlled trial design in future studies and Guise et al [33] who only performed initial usability testing for two narrative virtual patients that they developed for vocational mental health nurse training. McEvoy et al [34] examined virtual patients as an educational intervention to improve pediatric basic specialist trainee education in the management of suspected child abuse. Their evaluation methodology focused on the use of a questionnaire, developed to determine trainees’ perception of the value of the virtual patient as an educational tool, and it has not yet been evaluated by professionals.

### Research Evidence From Controlled Trials Supporting the Effectiveness of Serious Games in Medical Education

As shown in the previous section of this paper, there is an abundance of studies that involve virtual patients in serious games in medical education that focus on the description of the design and development of innovative ways to teach doctors and medical students. However, only a few of them refer to virtual patient games for emergency treatment in cardiology.

Studies that advanced to controlled trial designs or similar rigorous methodologies, involving random assignments to experimental and control groups, to research the effectiveness of proposed serious games interventions in medical education have not been numerous. Research evidence from controlled trials supports that serious games [9] and the deployment of virtual patients [11,13] offer the potential to enhance learning and improve subsequent performance when compared to traditional educational methods in areas such as in basic life support skills [11], knowledge acquisition about pediatric respiratory diseases [13], hematology and cardiology topics [14], and cardiac examination competency in medical students [15]. Research also showed that small duration (eg, 1 hour) interventions of virtual patient-based e-learning programs are not necessarily more effective compared to traditional training in the area of improving physicians’ substance abuse management skills [12]. There are also some studies in the literature that, even though they used a robust methodology, did not measure learning gains but focused on the effect of virtual patient training on students’ confidence instead, in areas such as history-taking and clinical breast examination [35,36].

Of interest for this study are controlled trial studies that focused on emergency treatment in cardiology through virtual patients and these studies are discussed next. In an area that partly relates to emergency cardiology incidents’ treatment, as it involves basic life support with the use of a defibrillator, Kononowicz et al [11] introduced a voluntary virtual patients’ module into a basic life support with an automated external defibrillator (BLS-AED) course to examine whether this addition would improve the knowledge and skills of students taking the course. Half of the students were randomly assigned to an experimental group and given voluntary access to a virtual patient module consisting of six cases presenting BLS-AED knowledge and skills. The study was conducted over 6 weeks and involved 226 first-year medical students. The voluntary module was used by 61 of the 114 entitled study participants. The group that used virtual patients demonstrated better results in knowledge acquisition and in some key BLS-AED action skills than the group without access, or those students from the experimental group deliberately not using virtual patients.

In a study that involves cardiology but not emergency treatment, positive results that indicate better retention with virtual patients than with traditional learning methods have also been reported by Botezatu et al [14], who conducted a randomized controlled study on early and delayed assessment results of 49 students using virtual patients for learning and examination of hematology and cardiology topics in an internal medicine course.

In the area of emergency treatment but not necessarily in cardiology, Dev et al [37] tested the architecture of a virtual emergency department patient for scenarios in emergency medicine in a multi-person learning environment based on online gaming technology. The efficacy of the model and the virtual emergency department learning environment was evaluated in a study where 12 advanced medical students and first-year residents managed six trauma cases, in groups of four. Their pre- and post-test performance results showed significant learning, with results comparable to those obtained in human mannequin simulators.

Other researchers did not focus on the comparison between virtual patients and traditional modules but rather tried to maximize the benefits of both approaches by combining them. These attempts demonstrated that virtual patients in virtual worlds or as part of serious games offer significant learning potential when used as a supplement to the traditional teaching techniques of medical education. Evidence for this comes from studies with virtual patients as a supplemental teaching tool for pediatric dentistry from Papadopoulos et al [10] and for clinical skills training for practicing health care workers Triola et al [23]. Similar studies in the areas of cardiology and emergency treatment were not found in the literature.

### Trends in the Literature on Serious Games Involving Virtual Patients

The evidence outlined above demonstrates not only medical students’ potential uptake of serious games for education and training but also the potential effectiveness and capability of serious games employing virtual patients to increase students’ learning in various domains of medical education, including a few studies in emergency treatment in cardiology, which is the focus of this study. However, the literature review of recent research studies in the area of virtual patient implementation in medical education shows two gaps. The first gap is that there is a lack of evaluation/impact measurements with actual professional groups; as in most of the existing studies, participants were typically medical students. Two exceptions to the studies described above are the work of Heinrichs et al [38] (2010) and the work of Salminen et al [24]. Heinrichs et al [38] attempted to determine whether a virtual emergency department, designed after Stanford University Medical Center’s Emergency Department was an effective clinical environment for training emergency department physicians and nurses for mass-casualty incidents. The participants of the study were professionals, more specifically 10 physicians with an average of 4 years of post-training experience, and 12 nurses with an average of 9.5 years of post-graduate experience. Similarly, in the work of Salminen et al [24], the virtual patient model that was developed to facilitate medical students’ reflective practice and clinical reasoning as well as the case created using the developed model were validated by a group of 10 experienced primary care physicians and then further improved by a work group of faculty involved in that medical program.

The second gap identified in the relevant literature is that not many serious games exist that focus on supporting medical students’ skills in responding to medical emergency situations to treat the symptoms of patients facing cardiovascular complications through telemedicine. Some of the scenario-based games that exist, for example the ones that have been developed and repurposed as part of the eViP virtual patients’ project in the area of “Cardiology, Emergency Medicine, ECG”, are based on mainly textual information presented to the user, even though some incorporate audio and video. The user is typically asked to make a diagnosis choice among given options in multiple choice form (eg, eViP Mr Horcek, developed by DecisionSim). Even though some of these games provide instant or enquiry-based feedback to the user, others do not and are linear in nature. An important limitation of these games is that they are not real-time games simulating realistic conditions of emergency care, in the sense that the user does not have any time pressure and is not typically required to solve the problem in a limited amount of time, as is the case in reality, where if medical professionals do not react promptly they may lose the patient. Another limitation is that the user is not necessarily in contact with a virtual patient all the time; he or she may only be interacting with on-screen textual instructions or choices rather than observing the virtual patient or the signals of an electrocardiogram (ECG) at any given time.

Another category of existing games are commercial games, such as MicroSim [39], which can provide resource-efficient, self-directed learning through the simulation of realistic patient scenarios to help learners develop decision-making and critical-thinking skills. In the MicroSim pre-hospital version, patient cases are set in a pre-hospital environment as well as in an ambulance. The learner has access to most of the tools and drugs that are available in an ambulance as well as the initial scene of the pre-hospital environment [39]. Another example of a serious game implementing an immersive virtual learning space, which was developed for training health care professionals in clinical skills is Pulse!! - The Virtual Clinical Learning Lab. In this game, graphics recreate a lifelike, interactive, virtual training environment in which civilian and military health care professionals practice clinical skills in order to better respond to injuries sustained during catastrophic incidents, such as combat or bioterrorism. The game is designed to support a range of the training needs nurses and medical professionals require [8]. However, commercial simulations, such as Microsim and Pulse!! typically involve a high cost to be made available to hospitals for medical education, and are not always research-validated.

This study attempts to address the two identified gaps by not only designing and developing the Virtual Emergency TeleMedicine (VETM) serious game, but also involving a number of professionals, who are at the same time stakeholders, specifically professional ambulance crew nursing personnel specializing in the treatment of emergency cases, in the evaluation of the educational potential of this game. The design and development phase of the VETM game is described in the next section, which is followed by a quantitative, professional feedback-informed evaluation of the game.

### An Example of a Serious Game for Medical Emergency Situations: Virtual Emergency Telemedicine (VETM)

#### The Rationale Behind VETM

The VETM game is a simulation-based serious game that was developed in response to calls to integrate serious games in medical education and training. The game was also developed to provide a learning environment that can be used as a supplement to traditional training in emergency situations and that is, at the same time, compatible with today’s medical students’ preferences toward new media and new learning technologies. The game makes use of data from an ECG and is designed and developed to train practicing doctors, medical students, or other health care professionals, such as nurses and paramedics, for problem-solving in real-life emergency clinical scenarios through a telemedicine system. Users of the game learn how to respond to medical emergency situations to assess and manage the symptoms of a virtual patient, who is located in an ambulance, through different scenarios involving cardiovascular complications. The game allows users to practice their skills while receiving immediate feedback by the system and virtual patient. The VETM game is based on principles of adult learning and problem-based learning, such as self-pacing, contextualization, and a hands-on approach in which the learner is an active participant [7] and employs popular gamification tactics to engage the users. More specifically, the game is based on scenarios, clear goals are provided to the user who is committed to achieve them, points are allocated for correct performance actions for reinforcement purposes, progress of the user in the game is visible to enable progress monitoring, and constant feedback is provided to the user through the experience, as suggested by [40].

The concept of the VETM game is based on a real system developed by the eHealth labs of the University of Cyprus and Frederick University. This work initially aimed at the development of an integrated portable medical device for emergency telemedicine. The system enables the transmission of critical biosignals (ECG, blood pressure, heart rate, oxygen saturation, temperature) and still images of the patient, from the emergency site to an emergency call center; thus enabling physicians to direct pre-hospital care in a more efficient way, improving patient outcome and reducing mortality. The system was designed in order to operate over several communication links such as mobile, satellite, ADSL [41,42]. The system has been continuously updated on the technological issues related to telecommunications and operating system environment. In addition to the ongoing work on the emergency telemedicine systems, the eHealth lab group is working on the transmission of real-time video as presented in [43-46].

#### Technical Specifications of VETM

With regard to the technical specifications, the VETM game implements a scripting language called Scribulance, which is a blend of C and Pascal. Scribulance is a user-friendly high-level scripting language developed specifically for the purpose of the game. It enables the creation of custom scenarios, which are currently text-based. The game was created using the programming language C# with XNA for the game. In order to support a high-level scripting language for developing the game’s scenarios, a scenario compiler was also created. For testing and evaluation purposes, several scripts that used the features of the scripting language were implemented to examine whether they worked properly and a detailed command-by-command debugging was conducted to examine that the flow of the code worked as intended [47,48].

#### Instructional Design of VETM

**Figure 1:**
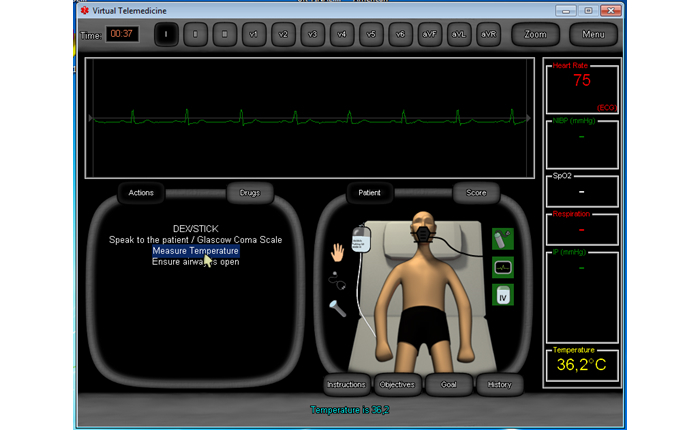
Screenshot of the Virtual Emergency TeleMedicine game that shows the interface with the virtual patient.

As Figure 1 shows, the interface of the game includes the “electrocardiogram” display with leads in the upper part, the “Actions” and “Drugs” available to the user in the left part, the virtual “Patient” in the right part, and additional information with regard to the physical state of the patient (heart rate, respiration, temperature, etc ) in the further right part of the screen. The “Actions” menu provides several options for the user to choose from. In this particular example, the user can “speak to the patient” to see if he is responding, “measure his temperature”, “ensure that his airway is open”, etc. The order of these options changes randomly every time the game is played and changes according to whether there are complications to the patient in the scenario. If the user chooses to provide drugs to the patient, then a drop-down menu is available under “Drugs” with several options of medication that are typically available in an ambulance (such as atropine sulphate, adrenaline, acetylsalicylic acid, paracetamol, salbutamol, hydrocortisone, etc) and the option to choose the dosage of each one.

Several “Tools” in graphical format are also provided. These are located around the patient. Tools include an “oxygen mask”, “providing IV fluids”, a “stethoscope”, the option to examine the patient with “palpations” or “perform Cardiopulmonary Resuscitation (CPR)”, a “flashlight” to examine the patient’s eye pupils, and the option to “enable the ECG” to monitor the patient’s heart (Figure 1).

After every one of the user’s actions (which can take place in one of three possible formats, either in the form of using a tool, or requesting an action, or providing medication), instant feedback is provided in the bottom part of the interface. In the example of Figure 1, the user attempted to measure the patient’s temperature and the feedback received was the following: “Temperature is 36.2 degrees Celsius”. The user receives a positive or negative score after each action. Users can check their score at any given time while playing the game. If they do, they get a detailed report of correct and incorrect actions up to that point. Alternatively, users can get a detailed report of actions that were correct (these are indicated in green color) and actions that were incorrect (these are indicated in red color) at the end of the game. The final report also includes the time needed to save the patient and the total score of the user.

As part of the instructional design of the game, the user can also access the specific “Objectives” for that scenario, the “Goals” of the game and the scenario’s “Instructions” that put the scenario into context. As shown in Figure 1, these are located below the virtual patient. In this scenario, named “Dyspnea and chest discomfort”, the “Instructions” were the following:

The Emergency Dispatch Centre received an emergency call and sent you to treat the following incident: “Husband feels bad, came home from work and is getting worse, dyspneic and with precordial pain”. You are the ambulance crew. You have to examine and treat the patient, define working diagnosis and differential diagnosis, administer the therapy, define direction according to local situation and possible following steps. Conditions on the scene: September 2, 2013, 13:30pm, clear sky, calm, temperature 20°C (68°F). Call to hospital is 10 minutes.

The user can also access and should consult the “History” of the patient prior to attempting to save him. In this scenario, the history of the patient that was available to the user was the following:

A 65-year-old man presented with dyspnea and chest discomfort. A week ago he had a similar episode, but symptoms have worsened now. Medical history: Lung cancer being treated with chemotherapy.

#### The Scenario-Editor of VETM

The game has two types of target populations: students and trainers. On the one hand, it can be used for training and practicing purposes in the context of given scenarios when the target population is users such as medical students, nurses, paramedics, and health professionals. On the other hand, it can be used for teaching or training purposes, when the target population is advanced users such as medical professors or people involved in training medical professionals, who can write scenarios of their own, with very limited prior knowledge of programming required, by using the scenario-editor of the game.

The Scenario Editor in VETM imports scenarios written in Scribulance, a very simple state-based scripting language similar to C. This language can describe a scenario in such a way that the choices and their effects are well-defined by the author. The scenario can then be added into the game easily. If there are no errors in the scenario, it will be added successfully and made available to play.

A medical professor or a trainer of medical professionals who is interested in adding a scenario to the game needs to know the basic structure of a scenario at a technical level, and this is described next. A Scenario is made up of States, Functions, and Variables.

A State may have any of the following Events: (1) a collection of Options that the player will see (an Option has a set of commands to be followed whenever that Option is selected), (2) an Enter Event (a set of commands to be followed whenever that State is activated), (3) an Exit Event (a set of commands to be followed whenever that State is deactivated), and (4) a collection of Time Events. For each Time Event, the user must specify a time in minutes and seconds. A Time Event is a set of commands to be followed when the specified time passes after the State has been activated.

Variables hold temporary values needed for a scenario. A variable can be, for example, the amount of times a patient was given a certain medication. A Function is a set of commands not associated to a State. It can have any number of Parameters (also known as Arguments) and may return a single value. Functions are typically used for code that is repeated multiple times in a Scenario. An Annotation is information, in text form, for one of the following: “History”, “Objectives”, and “Instructions”, which can be customized by the user in each scenario. A Scenario must have at least one State, while everything else is optional.

**Figure 2:**
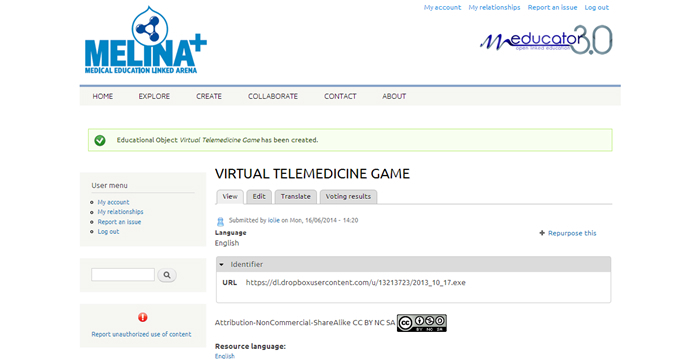
Interface of Melina+ (Medical Education Linked Arena), showing the Virtual Emergency TeleMedicine Game educational resource and metadata.

The VETM game is currently working as a standalone application on personal computers (PCs) with the Windows operating system [47,48]. The game can be shared and repurposed (through changing its scenarios, or creating new ones and compiling them with the included virtual emergency telemedicine compiler). It is discoverable through different instantiations of mEducator (Multi-type Content Sharing and Repurposing in Medical Education) [49], such as mEducator 3. 0 Melina+, an extended version of Drupal 7, which is offered as an installation profile and enables website administrators to install a learning management system, focused on medical education. As Figure 2 shows, the VETM game is listed there as a shared Internet resource, described with appropriate metadata.

### Research Question of the Study

The present study refers to the evaluation of one scenario (“Dyspnea and chest discomfort”) of the VETM game by professionals and attempts to provide an answer to the following research question: What are professionals’ perceptions of the potential of the Virtual Emergency TeleMedicine game for training people involved in treating emergency cases?

The “professionals” in the context of this study were professional ambulance crew ambulance crew nursing personnel specializing in assessing and managing pre-hospital emergency cases. The professionals’ “perceptions” referred to participants’ perceived advantages and limitations of the game. These were operationalized in the evaluation questionnaire that was developed under six dimensions of the game: (1) user interface, (2) difficulty level, (3) feedback, (4) educational value, (5) engagement, and (6) terminology.

## Methods

### Context of the Evaluation

The evaluation of the VETM game was conducted as part of a training seminar targeting professional ambulance crew nursing personnel specializing in assessing and managing pre-hospital emergency cases. The training was organized by the Cyprus Ministry of Health; it has a total duration of 250 hours spread over 2 years in about 50 five-hour meetings, and it is compulsory.

A total of 90 participants organized in four groups participated in the evaluation. The four training meetings took place on Oct. 6-7 and Oct. 13-14, 2014 in the computer lab of a pedagogical institute with 12 desktop computers. The duration of each meeting was approximately 1.5 hours. The evaluation sessions consisted of: (1) demo, (2) hands-on experience of the game, and (3) evaluation. The first part consisted of a brief 5-minute presentation of the game by the first author, to demonstrate the user interface, the tools that are available to the user, the ECG part, etc, and the signing of informed consent forms by participants that allowed the voluntary and anonymous use of their demographic data and questionnaire evaluation data, the audiotaping of their comments, and the examination of the log files that were automatically created when they interacted with the game. The participants were informed that they would be asked to complete a questionnaire to evaluate the game and to provide their comments verbally on a voluntary basis.

In the second part, the participants were asked to work in groups of two on each desktop computer to explore the game and to try to solve the scenario “Dyspnea and Chest Discomfort”. The scenario involved the examination, treatment, and diagnosis of a virtual patient who experienced dyspnea and precordial pain. The participants’ goal was dual: to solve the scenario correctly and also have a hands-on experience of the functionality of the game so that they could evaluate it. A total of 81 participants worked in groups and 9 participants worked as individuals. In 83% (75/90) of cases, participants worked in groups of two and in 7% (6/90) of cases, participants worked in groups of three.

In the third part, participants were asked to individually complete a questionnaire and also verbally express their thoughts via brief interviews on the improvement of the game, its value, if any, and points of confusion, etc, to the first author, who was audiotaping the participants’ responses.

### Participants

The participants of the study were 90 professional ambulance crew nursing personnel specializing in the assessment and management of pre-hospital emergency cases. With regard to ethical considerations, participation in the study was anonymous and voluntary, participants signed an informed consent form and the study protocol was previously approved by a review board at the University of Cyprus.

### Data Sources

#### Overview

The study included three data sources: a game evaluation questionnaire, the game’s automatically created log files, and participants’ comments in the form of short interviews, conducted either while they interacted with the game or immediately after. These data sources are described next.

#### Game Evaluation Questionnaire

As far as the description of the instrument for the evaluation of the game is concerned, this consisted of three parts. The first part consisted of demographic data such as: gender, age, previous experience with a telemedicine system, years of experience, domain of expertise, previous experience with educational games, and previous experience with medical education games.

The second part consisted of 36 questions on a 5-point Likert scale (Strongly disagree, Disagree, Neither agree nor disagree, Agree, Strongly agree). The instrument assessed six dimensions of the game: (1) user interface (8 questions, Q1-Q8); (2) difficulty level (5 questions, Q9-Q13); (3) feedback (5 questions, Q14-18); (4) educational value (9 questions, Q19-27); (5) user engagement (6 questions, Q28-Q33); and (6) terminology and language (3 questions, Q34-36).

An internal consistency reliability analysis was performed on these 36 items, which showed that the instrument appeared to have good internal consistency (Cronbach alpha=.9).

The third part of the questionnaire included 5 open-ended questions related to things that they liked or disliked in the game, things that were difficult or confusing, suggestions for improvement of the game, and whether they would be interested to use the game for training purposes. Users’ hand-written answers in the 5 open-ended questions of the questionnaire were typed and analyzed.

#### Game Log Files

Each time the game was played, a log file was automatically created with information on the time it took for the user to solve the scenario, the types of mistakes that he or she made, etc. The total number of log files that were analyzed was 379.

#### Users’ Audiotaped Comments From Short Interviews

Users’ audiotaped comments from short interviews were transcribed verbatim. A total of 24 short interviews were conducted with a total of 55 participants (40 male, 15 female) with a total duration of 106 minutes. Each brief interview lasted for an average of 4.4 minutes (minimum=1 minute, maximum=18 minutes).

### Analysis

With regard to the analysis of the responses of the questionnaire, data were coded in a statistical package (IBM/SPSS Statistics 20), using the numbers 1 to 5 for coding the answers (Completely Disagree to Completely Agree, respectively), and methods of descriptive analyses (main statistics of valid frequencies, means, and standard deviations) were calculated for all 36 questions. Four questions (Q10, Q12, Q17, Q20) were negatively phrased (eg, Q10 - The game is not challenging for me, Q12 - I needed more time to be able to solve the problem, Q17 - The feedback I receive when I make a choice is confusing, and Q20 - It was not clear what I could learn from the game) and their score was reversed prior to the calculation of compound scores. The calculation of compound scores was achieved as follows: the participants’ scores in the respective items under each one of the six dimensions that were examined were summed, and then recoded to reflect the 5-point Likert scale of the original questions. For example, for the second dimension, “difficulty level”, the sum of the five relevant questions (Q9-Q13) resulted in a possible minimum score of 5 and a maximum possible score of 25. This was recoded as follows: 5-8 were coded as value 1, 9-12 were coded as value 2, 13-16 were coded as value 3, 17-20 were coded as value 4, and 21-25 were coded as value 5. The same process was followed for all six dimensions, for consistency in reporting results.

The information contained in log files was transferred in SPSS for analysis. Overall, 23 different types of mistakes were coded and analyzed. The overall time for which all users in all four sessions engaged with playing the game was 13.5 hours.

The participants’ responses in the 5 open-ended questions of the questionnaire were coded for each question individually and frequencies of each code were calculated to give a sense of participants’ reactions to the game.

Users’ audiotaped comments from short interviews were analyzed qualitatively to complement themes from the analysis of the open-ended questions and to identify emerging themes of users’ perceptions on the VETM game. The users’ comments in the short interviews were initially classified in three broad categories corresponding to the areas that four out of five questions of the questionnaire examined: positive aspects of the game, negative or confusing aspects of the game, and suggestions for improvement. An additional theme that emerged examined the educational value of the game.

## Results

### Participants’ Demographic Information

The participants of the study were 90 professional ambulance crew nurses (67%, 60/90 male and 33%, 30/90 female). As can be seen from Table 1, participants’ average age was 32.23 years (SD 5.25, n=89) and it ranged from 25 to 49 years old. They had an average of 8.55 years of professional experience (mean 8.55, SD 5.26, n=85), which ranged from 1 to 26 years. The vast majority of participants 81% (71/90) had more than 5 years of experience, with 30% (27/90) of them having between 10 and 26 years of experience.

The vast majority of participants 87% (78/90) did not have any previous experience with games, either educational games or medical domain games. Of the people who had some experience with either type of game (13%, 12/89), their average time of playing games per week was approximately half an hour (0.57 hours per week). The majority of participants 70% (63/90) did not have any previous experience with a telemedicine system either.

**Table 1:** Participants’ demographic information (n=90).

	n	Mean	SD
Age (years)	89	32.23	5.25
Professional experience (years)	85	8.55	5.27
Game experience (hours per week)	70	0.57	2.17

### Users’ Behavior in Solving the Dyspnea and Chest Discomfort Problem

The analysis of log files showed that the game was played for a total of 379 times during the four evaluation sessions. Users attempted to solve the problem for an average of 8 times (mean 8.39, SD 3.6) with a minimum of 2 times and a maximum of 21. The result was “success”, which means that the patient was saved, in 20.6% (78/379) of these cases and the result was “failure”, which means that the virtual patient was lost, in 79.4% (301/379) of these attempts. The average time for which the users interacted with the game was 2.17 minutes at a time (SD 52.3 seconds) and it ranged from 15 seconds to 5 minutes for each time the scenario was played. Table 2 shows the frequencies of the 23 most common mistakes professionals made when attempting to solve the scenario Dyspnea and Chest Discomfort of the VETM game.

**Table 2:** Most common mistakes in solving the Dyspnea and Chest Discomfort scenario of the VETM (Virtual Emergency TeleMedicine) game (n=915).

Mistake number	Mistake description	Frequency n (%)
M4	Your patient is dying	301 (32.9)
M1	Eye pupils not checked	97 (10.6)
M3	Providing Adrenaline is inappropriate at this moment	92 (10.1)
M14	You have provided Oxygen before ensuring the airway was open	91 (9.9)
M12	The patient’s state has gotten worse!	59 (6.4)
M5	Providing Salbutamol is inappropriate at this moment	42 (4.6)
M8	CPR at this point was inappropriate	32 (3.5)
M6	Providing Hydrocortisone is inappropriate at this moment	30 (3.3)
M9	Temperature not checked	26 (2.8)
M7	Providing Atropine Sulphate is inappropriate at this moment	22 (2.4)
M10	Did not use DEX/STICK	20 (2.2)
M13	You turned the Oxygen off	20 (2.2)
M16	Providing Acetylsalicylic Acid is inappropriate at this moment	17 (1.9)
M2	Providing Furosemide is inappropriate at this moment	15 (1.6)
M19	Providing Nitroglycerin is inappropriate at this moment	11 (1.2)
M11	Did not attempt to interact with the patient	10 (1.1)
M17	Providing Glucagon is inappropriate at this moment	10 (1.1)
M18	Providing Nitrous Oxide is inappropriate at this moment	5 (0.6)
M15	Did not palpate the patient’s chest	4 (0.4)
M20	Providing Narcan is inappropriate at this moment	4 (0.4)
M23	ECG monitor not activated	4 (0.4)
M21	Providing Paracetamol is inappropriate at this moment	2 (0.2)
M22	Providing Morphine is inappropriate at this moment	1 (0.1)

### Users’ Evaluation of the VETM Game

Results of the evaluation are presented in Table 3 in two formats: (1) using frequencies (percentages of participants who fell into each one of the four categories ranging from “strongly disagree” to “strongly agree”), and (2) using descriptive statistics (means and standard deviations). Descriptive statistics are used to identify general trends in participants’ perceptions of the game and frequencies are used to examine the results of the participants’ response to each question in more detail (Table 3).

Table 4 summarizes the descriptive statistics of each one of the six dimensions evaluated in the VETM game (interface, feedback, difficulty level, educational value, engagement, and terminology). As can be seen from Table 4, overall results are relatively high with regard to users’ engagement (mean 3.99, SD 0.87, n=84), the game’s interface (mean 3.83, SD 1.0, n=83), and educational value (mean 3.83, SD 1.05, n=84), and a bit lower with regard to the feedback provided in the game (mean 3.4, SD 0.79, n=81), the difficulty level (mean 3.2, SD 0.65, n=84), and the terminology used in the game (mean 3.32, SD 1.0, n=84). The principal results from participants’ evaluation of the VETM game are further discussed in the next section of the paper.

**Table 3:** Results of the usability evaluation of the VETM (Virtual Emergency TeleMedicine) game through frequencies and descriptive statistics.

		Domain examined	n	Frequencies^a^					Descriptive statistics	
				S (%)	D (%)	N (%)	A (%)	SA (%)	mean	SD
**User interface (8 questions)**										
	1.	Accessing the game objectives was easy.	87	1.1	8.9	24.1	33.3	32.2	3.86	1.01
	2.	Accessing the game instructions was easy.	88	2.3	9.1	27.3	37.5	23.9	3.72	1.01
	3.	Accessing the patient history was easy.	88	3.4	3.4	15.9	37.5	39.8	4.07	1.00
	4.	The game is user-friendly.	87	3.4	6.9	13.8	37.9	37.9	4.00	1.06
	5.	I like the interface of the game.	87	4.6	5.7	19.5	41.4	28.7	3.84	1.06
	6.	The game graphics are adequate.	87	4.6	11.5	33.3	33.3	17.2	3.47	1.05
	7.	The response time of the game is as expected.	85	4.7	10.6	27.1	40.0	17.6	3.55	1.05
	8.	The game is easy to navigate.	86	1.2	19.8	15.1	37.2	26.7	3.69	1.11
**Difficulty level (5 questions)**										
	9.	Solving the problem was easy.	88	9.1	18.2	34.1	30.7	8.0	3.10	1.08
	10.	The game is not challenging for me.	87	17.2	25.3	25.3	16.1	16.1	2.89	1.32
	11.	The time allowed by the game for the doctor to save the patient is sufficient.	88	6.8	13.6	25.0	37.5	17.0	3.44	1.13
	12.	I needed more time to be able to solve the problem in Scenario Dyspnea and Chest Discomfort.	86	9.3	24.4	34.9	25.6	5.8	2.94	1.06
	13.	The game is complicated.	86	17.4	23.3	33.7	19.8	5.8	2.73	1.14
**Feedback (5 questions)**										
	14.	Keeping track of my score while playing the game was easy.	86	4.7	12.8	22.1	34.9	25.6	3.64	1.14
	15.	The game provides ways to recover after making a mistake.	85	29.4	14.1	29.4	21.2	5.9	2.6	1.27
	16.	The feedback I receive when I make a choice is adequate.	85	4.7	15.3	41.2	25.9	12.9	3.27	1.03
	17.	The feedback I receive when I make a choice is confusing.	86	8.1	12.9	46.5	22.1	10.5	3.14	1.04
	18.	I can learn from my mistakes when I play the game.	85	5.9	10.6	20.0	31.8	31.8	3.73	1.19
**Educational value (9 questions)**										
	19.	I learned how to diagnose and treat complications of … in the game.	87	9.2	9.2	26.4	39.1	16.1	3.44	1.15
	20.	It was not clear what I could learn from the game.	86	9.3	23.3	40.7	22.1	4.7	2.9	1.0
	21.	I found the game educational.	85	4.7	7.1	24.7	36.5	27.1	3.74	1.08
	22.	The game will be interesting for medical students.	87	3.4	6.9	17.2	39.1	33.3	3.92	1.05
	23.	The game will be useful for medical student training.	87	5.7	8.0	19.5	36.8	29.9	3.77	1.14
	24.	This game is a useful learning aid.	87	3.4	3.4	24.1	40.2	28.7	3.87	0.99
	25.	Learning objectives are clearly identified.	87	4.6	3.4	34.5	32.2	25.3	3.7	1.04
	26.	I would recommend the game to my colleagues.	87	5.7	4.6	25.3	37.9	26.4	3.75	1.08
	27.	If I were an instructor I would like to use the game in a classroom setting with my students.	87	6.9	8.0	16.1	37.9	31.0	3.78	1.18
**Engagement (6 questions)**										
	28.	I was motivated to undertake the challenge of the game.	86	2.3	9.3	23.3	46.5	18.6	3.7	0.96
	29.	I am interested in learning about how to react in cardiovascular emergency situations.	87	0.0	3.4	16.1	39.1	41.4	4.18	0.83
	30.	I feel “in control” when I play the game.	87	6.9	11.5	26.4	41.4	13.8	3.44	1.09
	31.	I was absorbed in the activity of the game.	87	2.3	3.4	27.6	44.8	21.8	3.8	0.9
	32.	I felt that time passed quickly.	86	2.3	3.5	22.1	43.0	29.0	3.93	0.93
	33.	The game was worthwhile.	85	2.4	2.4	22.4	41.2	31.8	3.98	0.93
**Terminology (2 questions)**										
	34.	The terminology used is correct.	85	4.7	12.9	31.8	36.5	14.1	3.42	1.04
	35.	The terminology used is consistent.	85	4.7	11.8	31.8	42.4	9.4	3.4	0.98
	36.	I would prefer the VETM game in Greek rather than English.	86	2.3	1.2	16.3	16.3	64.0	4.38	0.96

^a^SD=Strongly disagree, D=Disagree, N=Neither agree nor disagree, A=Agree, SA=Strongly agree.

**Table 4:** Descriptive statistics of the compound scores of the six dimensions of the game.

Dimensions of the game	n	Mean	SD
Interface	83	3.83	1.01
Feedback	81	3.40	0.79
Difficulty level	84	3.20	0.65
Educational value	84	3.83	1.05
User engagement	84	3.99	0.87
Terminology	84	3.32	1.00

### Users’ Attitudes Toward the Game

Users’ attitudes toward the game were examined using two qualitative data sources: the users’ written comments in the five questions of the questionnaire and the users’ verbal comments in brief interviews. Users’ audiotaped comments complemented data that came from the open-ended questions of the questionnaire and provided more extensive explanations for users’ reactions and attitudes toward the game. As negative comments and game features that users found confusing or difficult spontaneously led to suggestions for improvement, these are grouped together in the following section, which starts with users’ negative comments and naturally leads to their suggestions for improving the game.

### Users’ Identification of Confusing or Difficult Features of the Game and Suggestions for Improvement of the Game

Users identified features of the game that created confusion or hindered their problem-solving process while interacting with the game in Questions 2 and 3 and provided suggestions for improving the game in Question 4 of the questionnaire.

More specifically, Question 2 (What did you not like in the VETM game?) was answered by 57 people. One in five people (21%, 15/57) noted a few bugs that need to be improved, 8% (6/57) of people thought that the time allowed to save the patient especially when in state of complication was not adequate, and the same number of people disliked the fact that the game was written in the English language instead of the Greek language (8%, 6/57). Other negative features that were reported by only three people referred to the need to have a larger number of choices available (3/57), and the graphics (3/57) and colors of the game (3/57).

Question 3 (What things were difficult or caused confusion in the game?) was answered by 51 people; 28% (13/51) of people again referred to some bugs in the game, while 11% (5/51) of people said that it was not difficult so they didn’t have anything to report. Three people referred to other difficulties within the game such as providing the exact dosage of medication (3/51), the use of the English language (3/51), and difficulty in evaluating the state of the patient (3/51).

Question 4 (Do you have any suggestions for improving the game?) was answered by 57 people; 13% of people suggested collaboration with experienced nursing personnel to improve the game and correcting the bugs that were identified (8/57) and 8% suggested translating the game into Greek (5/57), adding options for possible interventions that nurses can take (3/57), and improving graphics (3/57).

With respect to negative or confusing aspects of the game as these were coded from the analysis of the users’ interviews, the following features were identified, at least once, by users who provided suggestions to alleviate difficulties: more closely following the “ABCDE” protocols with which nurses are familiar (M14, Interview 7; M26, Interview 13), using the generic names of drugs (Male Trainer, Interview 6), adding all possible drugs that could be administered, have them visible in all scenarios that are developed (F6, Interview 10; F7, Interview 11; M37, Interview 22; M38, Interview 23) and include the standardized dosage for each one (M21, Interview 10; M35, Interview 21), adding the heart, lungs, and ECG sounds to the game (M14, F15 Interview 7), making the time that passes when performing CPR more visible by increasing the font and changing the color of the written feedback (M15, Interview 8; M37, Interview 22; M39, Interview 23), provide the ability to give adrenaline while performing CPR (M22, Interview 10; M32, Interview 19; M37, Interview 22), having the defibrillator present at all times rather than making it appear only in scenarios where it is needed (M28, Interview 14), having the user chose current voltage and joules for defibrillation (M29, Interview 15), and last, translate the game into the Greek language as users are more familiar with the terminology in Greek rather than English (M28, Interview 14; M38, Interview 23).

An interesting aspect that emerged from the analysis of the interviews had to do with the desire of experienced users to have less guidance and less scaffolding in the game. Users commented that, if the target group is professionals, then the game should provide more advanced options (M7, Interview 4; M15, Interview 8; M23, Interview 11; M27, Interview 14; M39, Interview 23; F15, Interview 24) and not make these options immediately available to the user (M23, Interview 11). They also suggested additions of more advanced options, such as the addition of a greater number of types of oxygen masks (M23, Interview 11; M9, Interview 4; F2, Interview 5; F8, Interview 13) so that the user can learn how to identify the correct mask to be applied depending on each medical incident that is presented in the game and the addition of more advanced questions, such as “how much oxygen to provide” instead of simply providing oxygen (M9, Interview 4; F2, Interview 5), or “identify which type of IV should be induced” rather than simply providing IV for experienced users (M15, Interview 8; M14, Interview 7), or “getting a different sound or input when examining a different part of the lungs” (M16, Interview 8; M20, Interview 10). Experienced participants could, however, understand that the game in its current state would be useful for medical students and first-year nurses who are inexperienced. Another suggestion included changing the animated character of the virtual patient into a human character to make the game more realistic (F5, Interview 8).

## Discussion

### Principal Results

This study aimed to perform a professional feedback-informed evaluation of the VETM game to identify professionals’ perceptions of the game’s potential for training people involved in the assessment and management of emergency cases in cardiology. Overall, the results were positive in all six dimensions that were assessed: game interface, feedback, difficulty level, educational value, user engagement, and terminology used in the game (with means ranging from 3.2 to 3.99 out of 5 in the six dimensions). This finding indicates that professionals can see the potential of the VETM game for training, practicing, or evaluating users’ problem-solving skills in real-life clinical scenarios through a telemedicine system and a virtual patient and is in agreement with previous studies that documented positive attitudes of medical students on games [1], favorable attitudes of junior doctors on new media, and favorable attitudes of medical students on the use of serious games involving virtual patients in medical education [19].

Evaluation results for each dimension are discussed in more detail. The first dimension of the evaluation referred to the interface of the game and it was assessed based on the cumulative results of 8 relevant questions. As can be seen from Table 4, the compound mean score of the game’s interface was 3.83 (SD 1.01), which provides an indication that the interface of the game was satisfactory. As can be shown from more detailed results reported in Table 3, three out of four participants (76%, 66/87) thought that the game was user friendly (Q4), 70% (61/87) of them liked the interface of the game (Q5), and 64% (55/86) thought that the game was easy to navigate (Q8). More than 60% of participants agreed or strongly agreed that accessing the game objectives (Q1, 66%, 57/87) and instructions (Q2,61%, 54/88), and the patient’s history (Q3, 77%, 68/88) was easy. However, relatively lower scores were received for the response time of the game (Q7, mean 3.55, SD 1.05, n=85) and for the evaluation of the graphics of the game (Q6, mean 3.47, SD 1.05, n=87). The last point may relate to the suggestion made by three participants to improve the graphics of the game and the comment about changing the animated character to a human character in one of the interviews.

The second dimension of the evaluation referred to whether the difficulty level of the game was appropriate (mean 3.2, SD 0.65, n=84) and it was assessed based on the cumulative results of 5 relevant questions, two of which that were negatively phrased (Q10 and Q12) were reversed. More than half of participants (55%, 48/88) thought that the time allowed to solve the problem was sufficient (Q11, mean 3.44, SD 1.13, n=88). Almost 40% (34/88) of participants thought that solving the problem was easy, while 27% (24/88) of participants thought that solving the problem was difficult. It is important to note that around 34% (30/88) of participants were not sure whether the scenario they tried was easy or difficult. This is partly reflected by the success rate that was calculated from the analysis of 379 log files of the game that showed that participants tried to solve the problem for an average of 8 times and their success rate was generally low (20.6%). It is possible that participants’ personal characteristics such as previous professional experience, experience with a telemedicine system and experience with playing educational games may have influenced their perceptions with regard to the difficulty level of the game.

The third dimension of the evaluation referred to the adequacy of feedback provided by the game to support users’ learning (mean 3.40, SD 0.79, n=81) and it was assessed based on the cumulative results of 5 relevant questions. A total of 63% (54/85) thought that they could learn from their mistakes while playing the game (Q18). Keeping track of their score while playing the game was characterized as easy by 61% (52/86) of participants (Q14). The feedback received was characterized as adequate by 39% (33/85) of participants (Q16) and 27% (23/85) agreed that the game provides ways to recover after making a mistake (Q15). The last point relates to what seemed to be an area of misconception among participants who thought that the game should provide ways to recover after making any type of mistake as opposed to the design goal of the game to additionally simulate mistakes that are irreversible in real life and thus should be irreversible in the game environment as well.

The fourth dimension of the evaluation referred to the value of the game and it was assessed based on the cumulative results of 9 relevant questions. Results showed that participants greatly valued the VETM game (mean 3.83, SD 1.05, n=84). The majority of participants thought that the game is educational (64%, 54/85), it will be interesting for medical students (72%, 63/87), useful for medical student training (67%, 58/87), and useful as a learning aid (69%, 60/87). Even though the hands-on experience with playing the game was short, more than half of participants (55%, 48/87) thought that they learned how to diagnose and treat complications of cardiovascular type through the game and 57% (50/87) thought that the learning objectives were clearly identified. Last, 64% (54/85) of participants would recommend the game to their colleagues and 69% (60/87) would like to use it for teaching purposes. The last finding agrees with what participants also noted in their written comments in question 5 of the questionnaire and with what they reported in interviews, in which they referred to the originality of the game, they expressed an intention to download it to practice with more scenarios, and they referred to advantages such as the elimination of cost for training, the elimination of danger associated with dealing with real patients, and providing the ability for users to learn from their mistakes in a safe environment.

These findings are in accordance with what was reported in the literature with regard to the affordability, accessibility, and usability of serious games, reported by [9,23], and with regard to avoiding risks associated with real patients reported by [4,30]. Moreover, participants’ positive reaction toward the educational value of the game agrees with findings of previous studies that have been reported in the literature that documented medical students’ positive attitudes toward games [1-3,19].

The fifth dimension of the evaluation referred to engagement and it was assessed with 6 questions. It has the highest mean score of the six dimensions that were examined (mean 3.99, SD 0.87, n=84). The majority of participants felt that the game was worthwhile (73%, 62/85), that time passed quickly (72%, 62/86), they were absorbed in the activity (67%, 58/87), motivated to undertake the challenge of the game (65%, 56/86), and felt “in control” (55%, 48/87). Again, this finding also agrees with the generally positive attitudes of medical students and professionals toward games that were reported in the literature [1-3,19].

The sixth and last dimension of the evaluation referred to whether the terminology used in that particular scenario was correct and consistent (mean 3.32, SD 1.0, n=84) and it was assessed based on the cumulative results of two relevant questions.

As documented in the literature review, there is a lack of evaluation/impact measurements with actual professional groups, as most studies that aimed to evaluate serious games and virtual patients had students as participants. From a methodological standpoint, this study builds on the work of Heinrichs et al [38] who validated their work on a virtual emergency department with a small sample of professionals, specifically 22 participants.

Not many open source serious games exist that focus on supporting medical students’ skills in responding to medical emergency situations to manage the symptoms of patients facing cardiovascular complications through telemedicine. From a game design and development standpoint, this study overcomes some of the limitations of games developed as part of the eViP virtual patients’ project in the area of “cardiology, emergency medicine, ECG”. More specifically, as opposed to other non-commercial games that already exist in this area, the VETM game is not linear in nature, the order of choices provided to the user changes randomly each time the game is played, it is a real-time game simulating realistic conditions of emergency care where the user is required to solve the problem under time pressure, and it allows immediate contact with a virtual patient all the time as both the patient and signals of the ECG can be observed at any given time through the telemedicine system.

### Limitations

Even though the VETM game was validated by professionals with regard to its potential educational value in this study and results were positive, it has not yet been validated using a controlled trial design, which is part of the directions for future research in this area.

### Directions for Future Research

Most games for health care have not been validated as tools for education [7]. As Graafland et al [21] pointed out, games developed or used to train medical professionals need to be validated before they are integrated into teaching methods. Some weaknesses that were pointed out by participants will serve as input for the re-design of the VETM game. Further research should define valid performance parameters and formally validate any serious game before it can be seen as a fully-fledged teaching instrument for medical professionals. A direction of further research, to address the need for employing rigorous methodologies, such as controlled trials for the evaluation of virtual patients, will therefore be to determine the educational efficacy of VETM, in a controlled setting involving an experimental and a control group. The experimental group participants will interact with VETM as a way to practice their skills in emergency treatment while the control group participants will follow traditional teaching methods. Participants of both groups will be pre- and post-tested with respect to their problem-solving skills in treating a patient’s symptoms in an emergency situation.

Furthermore, the real emergency telemedicine system will be expanded with (1) the addition of emergency scenarios for patient handling [50]), (2) health care services and patient location management during major disasters [51], and (3) ultrasound emergency video transition from the ambulance to the hospital [44-46]. It is foreseen that the above functionality will be integrated in the VETM serious game for the support of advanced emergency telemedicine services.

### Conclusions

In response to calls to integrate serious games in medical education and training and in light of research findings that report the effectiveness of virtual patient implementation in medical education, this paper attempted to describe the design and evaluation of the Virtual Emergency TeleMedicine (VETM) game, a simulation-based virtual patient game that was developed in collaboration with the mEducator Best Practice network [49]. The VETM game makes use of data from an electrocardiogram to train practicing doctors or medical students for problem solving in real-life emergency clinical scenarios through a telemedicine system and virtual patients.

What makes the VETM game innovative and different from previous attempts to use telemedicine for training in emergency situations is that the game not only allows users to practice in the context of given scenarios but also allows them to write scenarios of their own with very limited knowledge of programming required, as the scenario editor uses a very simple state-based scripting language similar to C. Even though this functionality was not evaluated in the present study, it may provide a partial solution to the concern raised in the literature that refers to the difficulty and added costs for virtual patients to be authored, adapted, and exchanged. Another element that makes the VETM game different is the fact that it is made freely available through the Melina+ (Medical Education Linked Arena) platform for repurposing in different contexts, with different scenarios, in different languages, etc [49].

In response to the gap identified in the literature of a lack of evaluation/impact measurements with actual professional groups, this study followed a quantitative, professional feedback-informed evaluation of the educational potential of the VETM game. Professionals, who were also stakeholders in this case, included professional nurses, whose expertise is the assessment and management of emergency cases.

The results of the evaluation are promising with regard to the value of the game and provide a strong indication of the potential of this educational game in telemedicine. According to Cugelman [40], “users are the ultimate judges of intervention efficacy, so any gamified interventions will require user testing, to determine if they can work or not”(p. 4). Following Cugelman’s suggestion [40], the first users who tested the game were professionals, to provide their input and feedback as part of a quantitative evaluation of the game. This study showed that the inclusion of game elements in the domain of emergency treatment involving cardiovascular complications has the potential to enhance medical students’ learning experience and may increase their intrinsic motivation to practice, making the learning experience more enjoyable and potentially more effective, even though the latter remains to be seen. VETM can potentially become an attractive option for large-scale continuous medical education to help improve the knowledge of medical students, nurses, paramedics, rescuers, etc, on emergency treatment and potentially improve the diagnosis and treatment of patients who face cardiovascular or other problems.

## Abbreviations

BLS-AED: Basic Life Support Automated External Defibrillation
ECG: electrocardiogram
VETM: Virtual Emergency TeleMedicine
